# Supplementary material for: Resolving the full spectrum of human genome variation using Linked-Reads
Source: Genome Res. 2019 Apr;29(4):635–45. doi: 10.1101/gr.234443.118 (PMC6442396; doi:10.1101/gr.234443.118)
Supplement: Supplemental Material [file supp_gr.234443.118_Supplemental_longranger-2.2.zip › longranger-2.2.tar.bz2!/lr2.2-public/lib/stan/cmdstan/stan/doc/api/html/exponential_8hpp_source.html]

Stan: src/stan/prob/distributions/univariate/continuous/exponential.hpp Source File


|  |  |
| --- | --- |
|  | Stan  2.5.0  probability, sampling & optimization |


- Main Page
- Related Pages
- Namespaces
- Classes
- Files

- File List
- File Members

All Classes Namespaces Files Functions Variables Typedefs Enumerations Enumerator Friends Macros Pages

- src
- stan
- prob
- distributions
- univariate
- continuous

exponential.hpp

Go to the documentation of this file.

1 #ifndef STAN\_\_PROB\_\_DISTRIBUTIONS\_\_UNIVARIATE\_\_CONTINUOUS\_\_EXPONENTIAL\_HPP

2 #define STAN\_\_PROB\_\_DISTRIBUTIONS\_\_UNIVARIATE\_\_CONTINUOUS\_\_EXPONENTIAL\_HPP

3

4 #include <boost/random/exponential\_distribution.hpp>

5 #include <boost/random/variate\_generator.hpp>

6

7 #include <stan/agrad/partials\_vari.hpp>

8 #include <stan/math/error\_handling.hpp>

9 #include <stan/math/functions/value\_of.hpp>

10 #include <stan/meta/traits.hpp>

11 #include <stan/prob/constants.hpp>

12 #include <stan/prob/traits.hpp>

13

14 namespace stan {

15

16  namespace prob {

17

44  template <bool propto, typename T\_y, typename T\_inv\_scale>

45  typename return\_type<T\_y,T\_inv\_scale>::type

46  exponential\_log(const T\_y& y, const T\_inv\_scale& beta) {

47  static const char\* function = "stan::prob::exponential\_log(%1%)";

48

49  // check if any vectors are zero length

50  if (!(stan::length(y)

51  && stan::length(beta)))

52  return 0.0;

53

54  using stan::math::check\_positive\_finite;

55  using stan::math::check\_not\_nan;

56  using stan::math::check\_consistent\_sizes;

57  using stan::math::value\_of;

58

59  double logp(0.0);

60  check\_not\_nan(function, y, "Random variable", &logp);

61  check\_positive\_finite(function, beta, "Inverse scale parameter", &logp);

62  check\_consistent\_sizes(function,

63  y,beta,

64  "Random variable","Inverse scale parameter",

65  &logp);

66

67

68  // set up template expressions wrapping scalars into vector views

69  VectorView<const T\_y> y\_vec(y);

70  VectorView<const T\_inv\_scale> beta\_vec(beta);

71  size\_t N = max\_size(y, beta);

72

73  DoubleVectorView<

74  include\_summand<propto,T\_inv\_scale>::value,

75  is\_vector<T\_inv\_scale>::value> log\_beta(length(beta));

76  for (size\_t i = 0; i < length(beta); i++)

77  if (include\_summand<propto,T\_inv\_scale>::value)

78  log\_beta[i] = log(value\_of(beta\_vec[i]));

79

80  agrad::OperandsAndPartials<T\_y,T\_inv\_scale> operands\_and\_partials(y, beta);

81

82  for (size\_t n = 0; n < N; n++) {

83  const double beta\_dbl = value\_of(beta\_vec[n]);

84  const double y\_dbl = value\_of(y\_vec[n]);

85  if (include\_summand<propto,T\_inv\_scale>::value)

86  logp += log\_beta[n];

87  if (include\_summand<propto,T\_y,T\_inv\_scale>::value)

88  logp -= beta\_dbl \* y\_dbl;

89

90  if (!is\_constant\_struct<T\_y>::value)

91  operands\_and\_partials.d\_x1[n] -= beta\_dbl;

92  if (!is\_constant\_struct<T\_inv\_scale>::value)

93  operands\_and\_partials.d\_x2[n] += 1 / beta\_dbl - y\_dbl;

94  }

95  return operands\_and\_partials.to\_var(logp);

96  }

97

98  template <typename T\_y, typename T\_inv\_scale>

99  inline

100  typename return\_type<T\_y,T\_inv\_scale>::type

101  exponential\_log(const T\_y& y, const T\_inv\_scale& beta) {

102  return exponential\_log<false>(y,beta);

103  }

104

105

106

120  template <typename T\_y, typename T\_inv\_scale>

121  typename return\_type<T\_y,T\_inv\_scale>::type

122  exponential\_cdf(const T\_y& y, const T\_inv\_scale& beta) {

123

124  static const char\* function = "stan::prob::exponential\_cdf(%1%)";

125

126  using stan::math::check\_positive\_finite;

127  using stan::math::check\_nonnegative;

128  using stan::math::check\_not\_nan;

129  using boost::math::tools::promote\_args;

130  using stan::math::value\_of;

131

132  double cdf(1.0);

133  // check if any vectors are zero length

134  if (!(stan::length(y)

135  && stan::length(beta)))

136  return cdf;

137

138  check\_not\_nan(function, y, "Random variable", &cdf);

139  check\_nonnegative(function, y, "Random variable", &cdf);

140  check\_positive\_finite(function, beta, "Inverse scale parameter", &cdf);

141

142  agrad::OperandsAndPartials<T\_y, T\_inv\_scale>

143  operands\_and\_partials(y, beta);

144

145  VectorView<const T\_y> y\_vec(y);

146  VectorView<const T\_inv\_scale> beta\_vec(beta);

147  size\_t N = max\_size(y, beta);

148  for (size\_t n = 0; n < N; n++) {

149  const double beta\_dbl = value\_of(beta\_vec[n]);

150  const double y\_dbl = value\_of(y\_vec[n]);

151  const double one\_m\_exp = 1.0 - exp(-beta\_dbl \* y\_dbl);

152

153  // cdf

154  cdf \*= one\_m\_exp;

155  }

156

157  for(size\_t n = 0; n < N; n++) {

158  const double beta\_dbl = value\_of(beta\_vec[n]);

159  const double y\_dbl = value\_of(y\_vec[n]);

160  const double one\_m\_exp = 1.0 - exp(-beta\_dbl \* y\_dbl);

161

162  // gradients

163  double rep\_deriv = exp(-beta\_dbl \* y\_dbl) / one\_m\_exp;

164  if (!is\_constant\_struct<T\_y>::value)

165  operands\_and\_partials.d\_x1[n] += rep\_deriv \* beta\_dbl \* cdf;

166  if (!is\_constant\_struct<T\_inv\_scale>::value)

167  operands\_and\_partials.d\_x2[n] += rep\_deriv \* y\_dbl \* cdf;

168  }

169

170  return operands\_and\_partials.to\_var(cdf);

171  }

172

173  template <typename T\_y, typename T\_inv\_scale>

174  typename return\_type<T\_y,T\_inv\_scale>::type

175  exponential\_cdf\_log(const T\_y& y, const T\_inv\_scale& beta) {

176

177  static const char\* function = "stan::prob::exponential\_cdf\_log(%1%)";

178

179  using stan::math::check\_positive\_finite;

180  using stan::math::check\_nonnegative;

181  using stan::math::check\_not\_nan;

182  using boost::math::tools::promote\_args;

183  using stan::math::value\_of;

184

185  double cdf\_log(0.0);

186  // check if any vectors are zero length

187  if (!(stan::length(y)

188  && stan::length(beta)))

189  return cdf\_log;

190

191  check\_not\_nan(function, y, "Random variable", &cdf\_log);

192  check\_nonnegative(function, y, "Random variable", &cdf\_log);

193  check\_positive\_finite(function, beta, "Inverse scale parameter",

194  &cdf\_log);

195

196  agrad::OperandsAndPartials<T\_y, T\_inv\_scale>

197  operands\_and\_partials(y, beta);

198

199  VectorView<const T\_y> y\_vec(y);

200  VectorView<const T\_inv\_scale> beta\_vec(beta);

201  size\_t N = max\_size(y, beta);

202  for (size\_t n = 0; n < N; n++) {

203  const double beta\_dbl = value\_of(beta\_vec[n]);

204  const double y\_dbl = value\_of(y\_vec[n]);

205  double one\_m\_exp = 1.0 - exp(-beta\_dbl \* y\_dbl);

206  // log cdf

207  cdf\_log += log(one\_m\_exp);

208

209  // gradients

210  double rep\_deriv = -exp(-beta\_dbl \* y\_dbl) / one\_m\_exp;

211  if (!is\_constant\_struct<T\_y>::value)

212  operands\_and\_partials.d\_x1[n] -= rep\_deriv \* beta\_dbl;

213  if (!is\_constant\_struct<T\_inv\_scale>::value)

214  operands\_and\_partials.d\_x2[n] -= rep\_deriv \* y\_dbl;

215  }

216  return operands\_and\_partials.to\_var(cdf\_log);

217  }

218

219  template <typename T\_y, typename T\_inv\_scale>

220  typename return\_type<T\_y,T\_inv\_scale>::type

221  exponential\_ccdf\_log(const T\_y& y, const T\_inv\_scale& beta) {

222

223  static const char\* function = "stan::prob::exponential\_ccdf\_log(%1%)";

224

225  using stan::math::check\_positive\_finite;

226  using stan::math::check\_nonnegative;

227  using stan::math::check\_not\_nan;

228  using boost::math::tools::promote\_args;

229  using stan::math::value\_of;

230

231  double ccdf\_log(0.0);

232  // check if any vectors are zero length

233  if (!(stan::length(y)

234  && stan::length(beta)))

235  return ccdf\_log;

236

237  check\_not\_nan(function, y, "Random variable", &ccdf\_log);

238  check\_nonnegative(function, y, "Random variable", &ccdf\_log);

239  check\_positive\_finite(function, beta, "Inverse scale parameter",

240  &ccdf\_log);

241

242  agrad::OperandsAndPartials<T\_y, T\_inv\_scale>

243  operands\_and\_partials(y, beta);

244

245  VectorView<const T\_y> y\_vec(y);

246  VectorView<const T\_inv\_scale> beta\_vec(beta);

247  size\_t N = max\_size(y, beta);

248  for (size\_t n = 0; n < N; n++) {

249  const double beta\_dbl = value\_of(beta\_vec[n]);

250  const double y\_dbl = value\_of(y\_vec[n]);

251  // log ccdf

252  ccdf\_log += -beta\_dbl \* y\_dbl;

253

254  // gradients

255  if (!is\_constant\_struct<T\_y>::value)

256  operands\_and\_partials.d\_x1[n] -= beta\_dbl;

257  if (!is\_constant\_struct<T\_inv\_scale>::value)

258  operands\_and\_partials.d\_x2[n] -= y\_dbl;

259  }

260  return operands\_and\_partials.to\_var(ccdf\_log);

261  }

262

263  template <class RNG>

264  inline double

265  exponential\_rng(const double beta,

266  RNG& rng) {

267  using boost::variate\_generator;

268  using boost::exponential\_distribution;

269

270  static const char\* function = "stan::prob::exponential\_rng(%1%)";

271

272  using stan::math::check\_positive\_finite;

273

274  check\_positive\_finite(function, beta, "Inverse scale parameter",

275  (double\*)0);

276

277  variate\_generator<RNG&, exponential\_distribution<> >

278  exp\_rng(rng, exponential\_distribution<>(beta));

279  return exp\_rng();

280  }

281  }

282 }

283

284 #endif

traits.hpp

stan::prob::exponential\_ccdf\_log

return\_type< T\_y, T\_inv\_scale >::type exponential\_ccdf\_log(const T\_y &y, const T\_inv\_scale &beta)

**Definition:** exponential.hpp:221

stan::agrad::OperandsAndPartials::to\_var

T\_return\_type to\_var(double logp)

**Definition:** partials\_vari.hpp:138

stan::math::check\_positive\_finite

bool check\_positive\_finite(const char \*function, const T\_y &y, const char \*name, T\_result \*result)

**Definition:** check\_positive\_finite.hpp:12

error\_handling.hpp

stan::prob::exponential\_cdf\_log

return\_type< T\_y, T\_inv\_scale >::type exponential\_cdf\_log(const T\_y &y, const T\_inv\_scale &beta)

**Definition:** exponential.hpp:175

stan::length

size\_t length(const T &)

**Definition:** traits.hpp:159

stan::DoubleVectorView

DoubleVectorView allocates double values to be used as intermediate values.

**Definition:** traits.hpp:358

stan::prob::exponential\_rng

double exponential\_rng(const double beta, RNG &rng)

**Definition:** exponential.hpp:265

partials\_vari.hpp

stan::agrad::value\_of

T value\_of(const fvar< T > &v)

Return the value of the specified variable.

**Definition:** value\_of.hpp:16

constants.hpp

stan::is\_vector

**Definition:** traits.hpp:83

stan::agrad::OperandsAndPartials

A variable implementation that stores operands and derivatives with respect to the variable...

**Definition:** partials\_vari.hpp:76

traits.hpp

stan::return\_type::type

boost::math::tools::promote\_args< typename scalar\_type< T1 >::type, typename scalar\_type< T2 >::type, typename scalar\_type< T3 >::type, typename scalar\_type< T4 >::type, typename scalar\_type< T5 >::type, typename scalar\_type< T6 >::type >::type type

**Definition:** traits.hpp:406

stan::is\_constant\_struct

Metaprogram to determine if a type has a base scalar type that can be assigned to type double...

**Definition:** traits.hpp:57

stan::math::value\_of

double value\_of(const T x)

Return the value of the specified scalar argument converted to a double value.

**Definition:** value\_of.hpp:24

stan::prob::include\_summand

Template metaprogram to calculate whether a summand needs to be included in a proportional (log) prob...

**Definition:** traits.hpp:35

stan::agrad::OperandsAndPartials::d\_x2

VectorView< double \*, is\_vector< T2 >::value, is\_constant\_struct< T2 >::value > d\_x2

**Definition:** partials\_vari.hpp:83

stan::math::check\_nonnegative

bool check\_nonnegative(const char \*function, const T\_y &y, const char \*name, T\_result \*result)

**Definition:** check\_nonnegative.hpp:52

stan::math::check\_consistent\_sizes

bool check\_consistent\_sizes(const char \*function, const T1 &x1, const T2 &x2, const char \*name1, const char \*name2, T\_result \*result)

**Definition:** check\_consistent\_sizes.hpp:13

stan::max\_size

size\_t max\_size(const T1 &x1, const T2 &x2)

**Definition:** traits.hpp:191

stan::math::check\_not\_nan

bool check\_not\_nan(const char \*function, const T\_y &y, const char \*name, T\_result \*result)

Checks if the variable y is nan.

**Definition:** check\_not\_nan.hpp:56

value\_of.hpp

stan::agrad::OperandsAndPartials::d\_x1

VectorView< double \*, is\_vector< T1 >::value, is\_constant\_struct< T1 >::value > d\_x1

**Definition:** partials\_vari.hpp:82

stan::agrad::log

fvar< T > log(const fvar< T > &x)

**Definition:** log.hpp:15

stan::VectorView

VectorView is a template metaprogram that takes its argument and allows it to be used like a vector...

**Definition:** traits.hpp:275

stan::agrad::exp

fvar< T > exp(const fvar< T > &x)

**Definition:** exp.hpp:16

stan::prob::exponential\_cdf

return\_type< T\_y, T\_inv\_scale >::type exponential\_cdf(const T\_y &y, const T\_inv\_scale &beta)

Calculates the exponential cumulative distribution function for the given y and beta.

**Definition:** exponential.hpp:122

stan::prob::exponential\_log

return\_type< T\_y, T\_inv\_scale >::type exponential\_log(const T\_y &y, const T\_inv\_scale &beta)

The log of an exponential density for y with the specified inverse scale parameter.

**Definition:** exponential.hpp:46


---

[ Stan Home Page ]

*© 2011–2014,
Stan Development Team.*
